# The 5 C model and Mpox vaccination behavior in Germany: a cross-sectional survey

**DOI:** 10.1186/s12889-024-18489-8

**Published:** 2024-04-15

**Authors:** Philip Oeser, Julianna Grune, Jendrik Dedow, Wolfram Joachim Herrmann

**Affiliations:** Institute of General Practice and Family Medicine, Charité– Universitätsmedizin Berlin, corporate member of Freie Universität Berlin and Humboldt- Universität zu Berlin, Charitéplatz 1, 10117 Berlin, Germany

**Keywords:** Monkeypox/prevention and control, Monkeypox/epidemiology, Environment and public health/vaccination, Sexual and gender minorities, Cross-sectional studies

## Abstract

**Background:**

Due to the authorization of the Mpox vaccines, we aimed to identify determinants of the intention to get vaccinated, actively trying to receive vaccination, and for successfully receiving a vaccination in Germany employing the 5 C model of vaccination readiness.

**Methods:**

Data stem from a cross-sectional online survey that was available online from August 13, 2022 to August 31, 2022. To assess the influence of the 5 C Model on vaccination behavior, we conducted a multinomial logistic regression.

**Results:**

3,338 participants responded to the survey, with 487 already vaccinated and 2,066 intending to receive a vaccination. Confidence and collective responsibility were positively associated with intention to get vaccinated, while complacency was negatively correlated. A higher score on the calculation scale increased the odds of intention to receive vaccination but not with actively having tried to receive a vaccination. Fewer perceived constraints were associated with higher odds to be vaccinated. Patients in practices that focus on HIV treatment were more likely to intend to get vaccinated, to have tried to get vaccinated and to be vaccinated, regardless of indication. While level of education had no impact, having an indication to get vaccinated was a strong predictor of vaccination behavior in all groups.

**Conclusion:**

Future vaccination campaigns should aim to reduce specific constraints of the target group and make vaccines widely available in primary care institutions beyond HIV-focused practices.

**Supplementary Information:**

The online version contains supplementary material available at 10.1186/s12889-024-18489-8.

## Background

Since May 2022, an increase in travel-unrelated Mpox infection was registered worldwide and shortly thereafter, the World Health Organization (WHO) declared Mpox a public health emergency of international concern [1]. In July and August 2022, the European Medicines Agency (EMA) and the U.S. Food and Drug Administration (FDA) authorized the vaccines Imvanex (EU) and Jynneos (US), previously approved for the treatment of smallpox, to be used to protect adults from Mpox infection [2]. In Germany, the federal states received varying amounts of the vaccine and used different methods of distribution, such as distribution to practices focusing on HIV treatment, to public health services or solely to university hospitals [3].

Research on the intention to receive a vaccination against Mpox is still limited and focused on health care workers [4] or the general population of specific countries regardless of indication [5, 6]. In a cross-sectional survey from the United Kingdom during the Mpox outbreak, which was in large part answered by participants who did not define themselves as heterosexual, 86% of the participants reported that they would accept to get vaccinated [7]. In another survey on rural-urban differences on attitudes towards Mpox among 582 men who have sex with men (MSM) in the United States, 77.1% report to not been vaccinated against Mpox, and the study showed a disproportionate distribution towards urban participants in the vaccinated population, as well as a lower intention to get vaccinated in the rural population [8]. In a recent study among patients on pre-exposure prophylaxis (PrEP) or with an HIV-positive status from France, 33.6% of participants responded to be hesitant towards Mpox vaccination [9]. Vaccine hesitancy has been defined as a “delay in acceptance or refusal of vaccination despite availability of vaccination services” [10] and is a complex global phenomenon which has been further complicated by social media and disinformation on the internet [11]. Additionally, vaccine confidence reportedly declined with the start of the COVID-19 pandemic [12]. Factors that play a major role as psychological antecedents in an individual’s behavior towards vaccinations were summarized in the 5 C Model [13]. It consists of 15 items in 5 categories, namely confidence in vaccines, complacency, constraints, calculation, and feelings of collective responsibility.

In this study, we aimed to identify predictors of intention to receive a vaccination and for successfully receiving a vaccination by evaluating to which extent the factors of the 5 C model have an influence on vaccination behavior. Additionally, we examined the influence of level of education and of being a patient in a HIV practice on receiving a vaccination against Mpox.

## Materials and methods

We conducted a cross-sectional online survey on Mpox in Germany from August 13 to August 31, 2022, on risk factors, vaccination and treatment status. The survey and an introductory text on the subject were shared through snowball sampling with more than 60 organizations that focus on lesbian, gay, bisexual, transgender, queer, intersex, and asexual (LGBTQIA) persons, which then shared the survey link via their contacts. It was promoted in a German newspaper (Tagesspiegel) and through social media. Inclusion criteria were age 18 or older and living in Germany at the time of the survey. Participants did not receive any incentive for taking part in the survey. The survey was created using “SoSci survey”, a web-application for online surveys which runs on a university server.

In the questionnaire, we asked participants whether they have already received a vaccination against Mpox. When they had not yet received a vaccination, participants were asked if they had the intention to get a vaccination and whether they have actively tried to get a vaccination, but have not received one yet (i.e., specifically searched for practices or institutions that offer appointments for vaccinations). When participants reported that they had received an Mpox vaccination, we asked whether they had received the first dose or both doses, and where the vaccination took place (public health institution, practice focusing on HIV-treatment, general practitioner, other primary care practice, university hospital, other hospital).

The questionnaire contained sociodemographic questions (i.e. on gender identity, sexual orientation, living environment, work status and level of education) and questions on sexual behavior (number of sex partners, gender identity of last sex partner, sexual practices with the last sex partner, use of protection with the last sex partner), based on the German Health and Sexuality Survey (GeSiD) [14] and modified to account for LGBTQIA + persons. Regarding vaccination behavior, we used the German version of the long 5 C scale, consisting of 15 items in the five categories confidence, complacency, constraints, calculation, and collective responsibility. The individual items contained in the 5 C model can be found in the Supplement Material.

To assess the influence of the 5 C Model on vaccination behavior regarding Mpox, we conducted a multinomial logistic regression. Multinomial regression analysis was conducted in R Statistical Software (R version 4.2.2) using the mlogit package [15]. The dependent outcome variable of the regression model was vaccination behavior, a multinomial variable with four categories: (1) no intention to receive vaccination (reference category), (2) intention to receive a vaccination, but not having tried to receive vaccination at the time of the study, (3) intention to receive and having tried to receive vaccination, and (4) successfully received vaccination. When participants responded that they “definitely” or “if possible” want to get vaccinated, it was considered a positive dependent variable for category 2. When they additionally responded that they have already tried to get vaccinated in the follow-up question, it was considered a positive dependent variable for category 3.

Independent variables were the 5 C subscales Confidence, Complacency, Constraints, Calculation, and Collective Responsibility. “Confidence” refers to an individual’s trust in the efficacy of vaccines and in public authorities’ decisions regarding vaccines. “Complacency” describes an individual’s perception of a vaccine-preventable disease as a threat. “Constraints” describe (structural and psychological) obstacles in receiving a vaccination, and “Calculation” refers to deliberation on the usefulness of a specific vaccine. “Collective Responsibility” is comprised of the willingness to protect others and to create herd immunity. The 5 C Model has been used in studies to examine attitude towards vaccines in different demographics and for different infectious diseases, such as COVID-19, measles, or influenza [16–18]. The categories of the 5 C scale showed a good internal consistency in a previous study (confidence α = 0.87, complacency α = 76, constraints α = 0.85, calculation α = 0.78, collective responsibility α = 0.71) [13]. While it has recently been extended into a 7 C Model, adding the categories conspiracy and compliance [19], the 5 C model is still the most used. The 5 C subscales were mean values of each subscale ranging from 1 (lowest) to 7 (highest). Another independent variable was the indication for receiving a vaccination. In our study, participants had an indication for vaccination when they did not define as women (i.e., defined their gender as men or non-binary), had sex with more than one person in the last 12 months and self-identified either as homosexual, gay, bisexual, or pansexual, or their last sexual partner identified either as a man or a non-binary person. While the official recommendations to get vaccinated also encompassed the risk of exposure to Mpox in a laboratory setting, we opted not to incorporate this aspect into our survey and consequently omitted it from our definition of “indication”.

To be a patient in a HIV clinic was added as independent variable, because in many federal states the vaccines were mainly distributed by means of these clinics. General Practitioners (GPs) are usually distributors of vaccines in Germany, thus we included whether the participant had a GP as another variable. In a previous study on risk factors of Mpox infection based on the same survey, results showed that a lower level of education was associated with higher odds for being infected [20], education level was added as another independent variable. For each subscale of the 5 C Model, we calculated the mean response as a score for each participant. All participants were digitally asked for consent before their participation and the survey was reviewed and approved by the Ethics Committee of Charité– Universitätsmedizin Berlin (EA1/180/22).

## Results

**Fig. 1:**
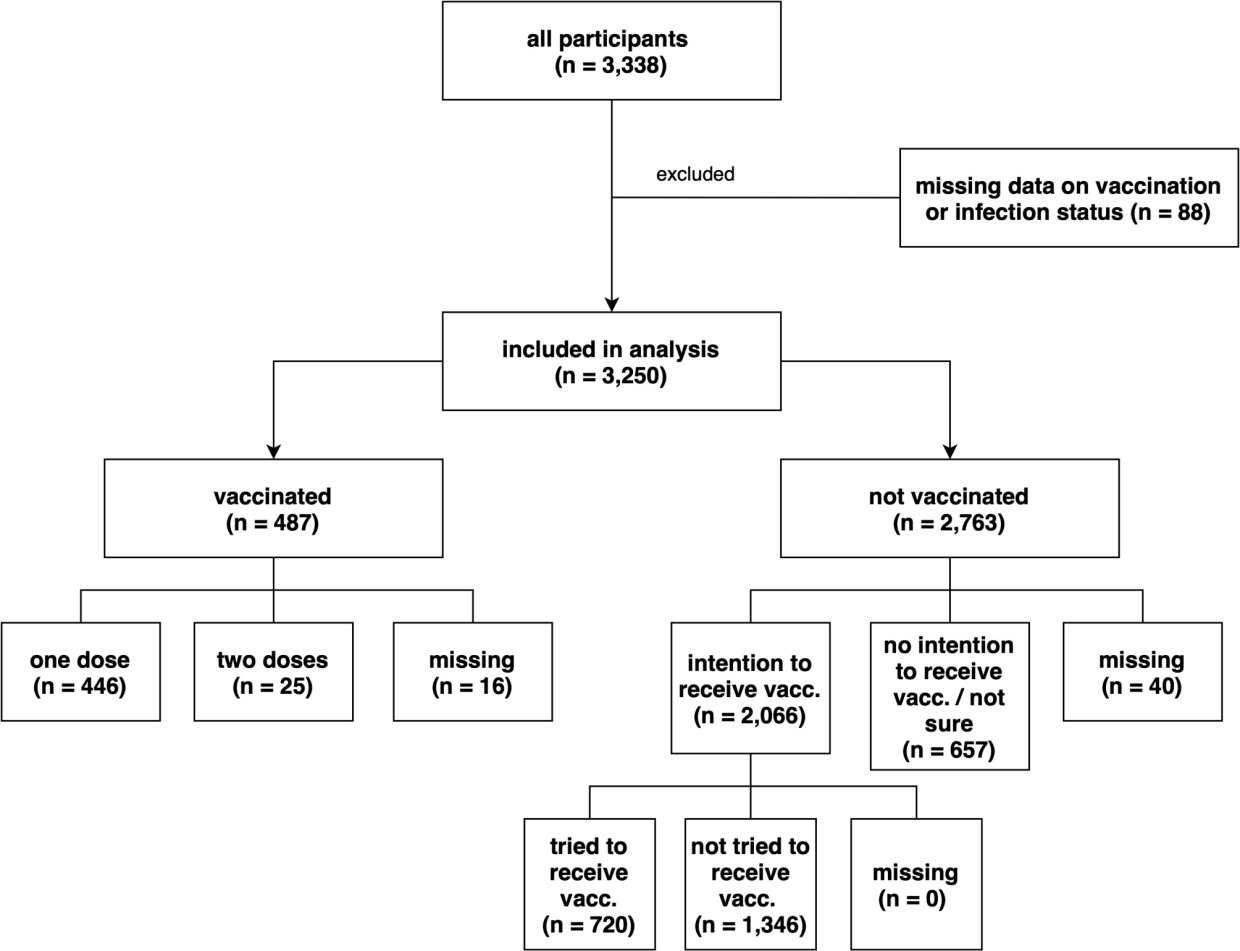
Participant flowchart

3,338 participants responded to the survey. 88 participants did not answer the question on vaccination status, and were excluded from the analysis. Of the 3,250 remaining participants, 487 were vaccinated, with 446 (13.7%) reporting to have received one dose at the time of the survey, while 25 (0.8%) had received both doses. 2,066 (74.7%) of the 2,763 unvaccinated participants answered had the intention to receive a vaccination, and of those, 720 (34.8%) actively tried to, but had not yet received vaccination at the time of the study (cf. Fig. 1).

178 (6.4%) participants answered that they would not want to get vaccinated against Mpox, and 479 (17.3%) were still unsure, or had not thought about their intent before participating in the survey. 40 participants did not respond whether they would like to receive vaccination. Of all included participants, 1,325 (40.8%) had an indication for an Mpox vaccination. Of those, 32 (3.5%) did not want to get vaccinated, and 79 (8.7%) were unsure or have not thought about receiving vaccination.

Table 1 shows the sociodemographic data of all included participants in total, and the groups of the regression analyses. Overall, most participants were between 26 and 45 years old, lived in an urban environment with 100,000 inhabitants or more and had an A-level education or higher. 614 participants (18.9%) identified as cis-heterosexual.

Distribution of participants’ answers of the 5 C questionnaire is shown in the Supplement Table.

**Table 1:** Demographic data of included participants, August 2022, n = 3,250

	All (n = 3,250)	No intention to receive vacc. (n = 657)	Intention to receive vacc. (n = 2,066)		Vaccinated (n = 487)
			Not tried to receive vacc. (n = 1,346)	Tried to receive vacc. (n = 720)	
**Age Group**					
18–25 years	423 (14.6%)	111 (19.4%)	226 (18.6%)	69 (10.5%)	17 (3.8%)
26–35 years	897 (31.0%)	171 (29.9%)	389 (32.1%)	228 (34.5%)	109 (24.5%)
36–45 years	818 (28.3%)	125 (21.9%)	308 (25.4%)	202 (30.6%)	183 (41.1%)
46–55 years	522 (18.1%)	100 (17.5%)	208 (17.1%)	119 (18.0%)	95 (21.3%)
56 or more years	229 (7.9%)	64 (11.2%)	82 (6.8%)	42 (6.4%)	41 (9.2%)
Missing Data	361	86	133	60	42
**Living Environment** ^†^					
Urban	2,136 (74.1%)	363 (63.7%)	829 (68.5%)	551 (83.6%)	393 (88.7%)
Middle sized town	338 (11.7%)	88 (15.4%)	173 (14.3%)	54 (8.2%)	23 (5.2%)
Small town	235 (8.2%)	63 (11.1%)	122 (10.1%)	38 (5.8%)	12 (2.7%)
Rural	173 (6.0%)	56 (9.8%)	86 (7.1%)	16 (2.4%)	15 (3.4%)
Missing Data	368	87	136	61	44
**Education**					
A-level or higher	2,551 (88.6%)	498 (87.5%)	1,060 (87.7%)	594 (90.1%)	399 (90.3%)
Under A-level	328 (11.4%)	71 (12.5%)	149 (12.3%)	65 (9.9%)	43 (9.7%)
Missing Data	371	88	137	61	45
**Identity / Orientation**					
Cis-heterosexual	614 (21.3%)	227 (39.9%)	354 (29.2%)	30 (20.5%)	3 (0.7%)
LGBTQIA+	2,271 (78.7%)	342 (60.1%)	858 (70.8%)	630 (95.5%)	441 (99.3%)
Missing Data	365	88	134	60	43

LGBTQIA+: lesbian, gay, bisexual, transgender, queer, intersex, asexual or other non-cis-heterosexual identity/orientation; ^†^ urban = > 100,000 inhabitants, middle sized town = > 20,000–100,000 inhabitants, small town = > 5,000–20,000 inhabitants, rural = up to 5,000 inhabitants

Results of the multinomial regression analysis can be found in Table 2. Confidence and collective responsibility were positively associated with the intention to receive a vaccination, actively trying to receive vaccination, and with being vaccinated at the time of the survey. Complacency was negatively associated in all groups; for example, with each increase of one point on the complacency scale, the odds of having actively tried to receive vaccination decreased by a factor of 0.6.

**Table 2:** Multinomial regression analysis

	Intention to receive vaccination (n = 2,066)		Vaccinated (n = 487)
	Not tried to receive vacc. (n = 1,346)	Tried to receive vacc. (n = 720)	
	**OR [95% CI]**	**OR [95% CI]**	**OR [95% CI]**
Intercept	0.02 [0.00, 0.08]	0.00 [0.00, 0.01]	0.00 [0.00, 0.02]
Confidence	**1.34 [1.15, 1.56]**	**1.37 [1.14, 1.65]**	**1.40 [1.12, 1.74]**
Complacency	**0.67 [0.57, 0.78]**	**0.60 [0.50, 0.72]**	**0.78 [0.63, 0.97]**
Constraints	**1.25 [1.14, 1.37]**	**1.30 [1.16, 1.45]**	**0.81 [0.70, 0.93]**
Calculation	**0.84 [0.77, 0.91]**	0.95 [0.86, 1.06]	0.97 [0.86, 1.10]
Collective Responsibility	**1.69 [1.39; 2.05]**	**1.87 [1.58, 2.23]**	**1.32 [1.06, 1.63]**
Indication^†^	**1.88 [1.42; 2.50]**	**11.56 [8.47, 15.78]**	**38.24 [24.05, 60.80]**
Level of Education	0.84 [0.60; 1.17]	1.15 [0.76, 1.75]	1.37 [0.83, 2.26]
Patient of HIV practice	1.42 [0.87, 2.31]	**4.67 [2.91, 7.50]**	**11.00 [6.76, 17.90]**
Patient of General Practice	0.97 [0.73, 1.30]	1.07 [0.75, 1.53]	0.76 [0.50, 1.18]

significant findings are highlighted in bold. CI: confidence interval, OR: odds ratio

^†^ Indication: persons not defining as women who self-identified as homosexual, gay, bisexual, or pansexual, with more than one sexual partner in the last 12 months

Calculation was positively associated with the intention to get vaccinated. Having an indication was positively associated with higher odds of intention without having tried, with actively having tried to receive a vaccination and for being vaccinated; for example, having an indication increased the odds of having actively tried to get vaccinated by a factor of 11.56, and the odds of being vaccinated by a factor of 38.24.

Constraints negatively impacted the intention as well as being vaccinated. For example, an increase of one point on the constraints scale decreased the odds to be vaccinated by 0.81, therefore the likelihood to be vaccinated was lower for participants who perceived higher constraints. It was more likely for participants to try to receive a vaccination and for being vaccinated when they were patients in a HIV practice. Level of education had no impact on vaccination behavior.

## Discussion

In our analysis on vaccination behavior regarding Mpox vaccinations in Germany, we could show that for the items of the 5 C Model, confidence and feelings of collective responsibility were positively associated with the intention to get vaccinated, while complacency had a negative influence. Calculation played a role in the intention, but not in actively trying to get vaccinated. When participants were patients of an HIV practice, it was more likely for them to have actively tried to get vaccinated or to already be vaccinated against Mpox.Perceived constraints made it less likely to be vaccinated. Having an indication for a vaccination was a strong predictor in all groups.

In our study, the overall intention to get vaccinated was lower compared to a study from the United Kingdom with mostly LGBTQIA + participants, in which 86% reported they would accept a vaccine. In their sample, non-MSM were less likely to accept a vaccine if offered, and the overall intention decreased with lower ability to afford basic needs and with lower education [7]. This stood in contrast to our results, where level of education had no influence on vaccination behavior.

Comparing our results to a study on the US general public, in which only 46% of respondents intended to get vaccinated even when it was recommended to them [6], our study showed a higher intention to receive a vaccination against Mpox. The study indicates that lack of clear communication might be a reason for the low intention to get vaccinated. The higher intention in our study can be attributed to the oversampling of LGBTQIA + participants, and as a potentially vulnerable group they might be more accepting of an Mpox vaccine. This is also in line with the recommendation to give the vaccine only to specific target demographics.

In a French study on Mpox vaccinations in MSM on PrEP or with a HIV positive status, 33.6% were hesitant to be vaccinated, but vaccination acceptance rose with number of sexual partners during the previous months [9]. This is in accordance with our findings, in which a higher number of sexual partners was a strong predictor in vaccination behavior in all groups.

In a study on COVID-19 vaccination behavior in students from Belgium, the Netherlands and Portugal, collective responsibility as well as confidence were most strongly related to a vaccination intention, concluding that characteristics associated with these factors should be targeted to improve vaccination campaigns. Constraints played a lesser role, although high self-efficacy had a decreasing effect on perceived constraints [17]. Our results also suggest confidence in vaccines as an influencing factor. It could be argued that confidence played a larger role in the COVID-19 pandemic, since the mRNA vaccines against COVID-19 that were more commonly used in Germany are still relatively new and it has been shown that post-pandemic, confidence in vaccines in general was described to have decreased [12].

Constraints in receiving a vaccination are associated with perceived subjective factors like everyday stress and feeling uncomfortable at a doctor’s office, but also with objective factors like a lack of access to vaccines and lack of service delivery [13]. In our results, decrease in perceived constraints was observed when participants successfully received a vaccination. These findings indicate that constraint might be a malleable factor in the vaccination behavior for Mpox vaccines.

Our study has several limitations. Due to the oversampling of LGBTQIA + participants, our study does not reflect the general population: people more interested in Mpox and in vaccinations in general might have had a higher probability of taking part in our study, however, we assume the subgroups to be comparable with each other. As our study was designed as a survey, cross-sectional data has limitations for causal relations. This is especially relevant in vaccination studies, as the uptake of a vaccination can influence the attitude towards vaccinations. Vaccination status was self-reported and might lead to misclassification. We also acknowledge that an online survey comes with a potential selection bias, as it requires internet access, technical understanding, and literacy skills. To account for better accessibility, the survey was offered in English and German, however, less than 30 participants accessed the English version. People with higher level of education were more likely to participate.

Missing data in our calculations are attributed to participants who quit the survey mainly on the first two pages.

## Conclusion

The findings of our study suggest that future vaccination campaigns against Mpox should focus on decreasing barriers, for example by making the vaccines widely available in primary care institutions, other than HIV-focused practices in sufficient amounts, thus allowing more people to follow through on their intention to get vaccinated. Public campaigns should be tailored to address the target groups’ specific perceived constraints and appeal to their feeling of collective responsibility.

### Electronic supplementary material

Below is the link to the electronic supplementary material.

Supplementary Material 1

## Data Availability

Due to the sensible content and informed consent, datasets used and analysed are not openly available. If in line with informed consent given, parts of the dataset can be made available from the corresponding author on reasonable request.
