# Supplementary material for: The 5 C model and Mpox vaccination behavior in Germany: a cross-sectional survey
Source: BMC Public Health. 2024 Apr 15;24:1039. doi: 10.1186/s12889-024-18489-8 (PMC11017625; doi:10.1186/s12889-024-18489-8)
Supplement: Supplementary file 1 — Supplementary Material 1 [file 12889_2024_18489_MOESM1_ESM.docx]

Supplement. Items of the long 5C scale and the answers of participants, August 2022, n = 3,250.

|  | **Strongly disagree [1]** | **Disagree [2]** | **Somewhat disagree [3]** | **Neutral [4]** | **Somewhat agree [5]** | **Agree [6]** | **Strongly agree [7]** | **Missing Data** |
| --- | --- | --- | --- | --- | --- | --- | --- | --- |
| **Confidence** | | | | | | | | |
| **“I am completely confident that vaccines are safe.”** | | | | | | | | |
| All (n = 3,250) | 29 (1.0 %) | 15 (0.5 %) | 33 (1.1 %) | 55 (1.8 %) | 278 (9.2 %) | 1,150 (38.0 %) | 1,468 (48.5 %) | 222 |
| No intention to receive vaccination (n = 657) | 20 (3.3 %) | 14 (2.3 %) | 28 (4.6 %) | 37 (6.0 %) | 107 (17.5 %) | 234 (38.2 %) | 173 (28.2 %) | 44 |
| Intention, not tried to receive vaccination (n= 1,346) | 3 (.2 %) | 0 (-) | 2 (.2 %) | 6 (.5 %) | 85 (6.6 %) | 521 (40.7 %) | 662 (51.8 %) | 67 |
| Intention, tried to receive vaccination (n = 720) | 1 (.1 %) | 0 (-) | 1 (.1 %) | 8 (1.2 %) | 48 (7.1 %) | 229 (33.7 %) | 392 (57.7 %) | 41 |
| Vaccinated (n = 487) | 5 (1.1 %) | 1 (.2 %) | 2 (.4 %) | 4 (.9 %) | 38 (8.3 %) | 166 (36.3 %) | 241 (52.7 %) | 30 |
| **“Vaccinations are effective.”** | | | | | | | | |
| All | 15 (.5 %) | 11 (.4 %) | 7 (.2 %) | 45 (1.5 %) | 170 (5.6 %) | 866 (28.6 %) | 1914 (63.2 %) | 222 |
| No intention to receive vacc. | 11 (1.8 %) | 7 (1.1 %) | 4 (.7 %) | 35 (5.7 %) | 74 (12.1 %) | 197 (32.1 %) | 285 (46.5 %) | 44 |
| Intention, not tried to receive vacc. | 2 (.2 %) | 0 (-) | 0 (-) | 5 (.4 %) | 43 (3.4 %) | 356 (27.8 %) | 873 (68.3 %) | 67 |
| Intention, tried to receive vacc. | 1 (.1 %) | 1 (.1 %) | 1 (.1 %) | 2 (.3 %) | 26 (3.8 %) | 177 (25.7 %) | 471 (68.5 %) | 41 |
| Vaccinated | 1 (.2 %) | 3 (.7 %) | 2 (.4 %) | 3 (.7 %) | 27 (5.9 %) | 136 (29.8 %) | 285 (62.4 %) | 30 |
| **“Regarding vaccines, I am confident that public authorities decide in the best interest of the community.”** | | | | | | | | |
| All | 74 (2.4 %) | 90 (3.0 %) | 211 7.0 %) | 265 (8.8 %) | 718 (23.7 %) | 937 (30.9 %) | 733 (24.2 %) | 222 |
| No intention to receive vacc. | 40 (6.5 %) | 22 (3.6 %) | 44 (7.2 %) | 47 (7.7 %) | 153 (25.0 %) | 189 (30.8 %) | 118 (19.2 %) | 44 |
| Intention, not tried to receive vacc. | 11 (.9 %) | 29 (2.3 %) | 95 (7.4 %) | 129 (10.1 %) | 333 (26.0 %) | 388 (30.3 %) | 294 (23.0 %) | 67 |
| Intention, tried to receive vacc. | 14 (2.1 %) | 23 (3.4 %) | 52 (7.7 %) | 64 (9.4 %) | 145 (21.4 %) | 201 (29.6 %) | 180 (26.5 %) | 41 |
| Vaccinated | 9 (2.0 %) | 16 (3.5 %) | 20 (4.4 %) | 25 (5.5 %) | 87 (19.0 %) | 159 (34.8 %) | 141 (30.9 %) | 30 |
| **Complacency** | | | | | | | | |
| **“Vaccination is unnecessary because vaccine-preventable diseases are not common anymore.”** | | | | | | | | |
| All | 2,414 (79.7 %) | 460 (15.2 %) | 91 (3.0 %) | 33 (1.1 %) | 12 (.4 %) | 6 (.2 %) | 11 (.4 %) | 223 |
| No intention to receive vacc. | 396 (64.7 %) | 124 (20.3 %) | 54 (8.8 %) | 21 (3.4 %) | 9 (1.5 %) | 2 (.3 %) | 6 (1.0 %) | 45 |
| Intention, not tried to receive vacc. | 1,078 (84.3 %) | 171 (13.4 %) | 23 (1.8 %) | 3 (.2 %) | 1 (.1 %) | 1 (.1 %) | 2 (.2 %) | 67 |
| Intention, tried to receive vacc. | 570 (83.9 %) | 93 (13.7 %) | 10 (1.5 %) | 3 (.4 %) | 0 (-) | 1 (.1 %) | 2 (.3 %) | 41 |
| Vaccinated | 370 (81.0 %) | 72 (15.8 %) | 4 (.9 %) | 6 (1.3 %) | 2 (.4 %) | 2 (.4 %) | 1 (.2 %) | 30 |
| **“My immune system is so strong; it also protects me against diseases.”** | | | | | | | | |
| All | 1,332 (44.0 %) | 694 (22.9 %) | 343 (11.3 %) | 360 (11.9 %) | 192 (6.3 %) | 88 (2.9 %) | 18 (.6 %) | 223 |
| No intention to receive vacc. | 205 (33.5 %) | 135 (22.1 %) | 95 (15.5 %) | 92 (15.0 %) | 48 (7.8 %) | 28 (4.6 %) | 9 (1.5 %) | 45 |
| Intention, not tried to receive vacc. | 655 (51.2 %) | 293 (22.9 %) | 122 (9.5 %) | 114 (8.9 %) | 66 (5.2 %) | 25 (2.0 %) | 4 (.3 %) | 67 |
| Intention, tried to receive vacc. | 309 (45.5 %) | 157 (23.1 %) | 72 (10.6 %) | 95 (14.0 %) | 32 (4.7 %) | 14 (2.1 %) | 0 (-) | 41 |
| Vaccinated | 163 (35.7 %) | 109 (23.9 %) | 54 (11.8 %) | 59 (12.9 %) | 46 (10.1 %) | 21 (4.6 %) | 5 (1.1 %) | 30 |
| **“Vaccine-preventable diseases are not so severe that I should get vaccinated.”** | | | | | | | | |
| All | 2,132 (70.5 %) | 631 (20.9 %) | 143 (4.7 %) | 67 (2.2 %) | 21 (.7 %) | 16 (.5 %) | 16 (.5 %) | 224 |
| No intention to receive vacc. | 329 (53.8 %) | 154 (25.2 %) | 61 (10.0 %) | 39 (6.4 %) | 15 (2.5 %) | 8 (1.3 %) | 6 (1.0 %) | 45 |
| Intention, not tried to receive vacc. | 970 (75.9 %) | 238 (18.6 %) | 45 (3.5 %) | 14 (1.1 %) | 2 (.2 %) | 2 (.2 %) | 7 (.5 %) | 68 |
| Intention, tried to receive vacc. | 505 (74.4 %) | 141 (20.8 %) | 20 (2.9 %) | 6 (.9 %) | 2 (.3 %) | 4 (.6 %) | 1 (.1 %) | 41 |
| Vaccinated | 328 (71.8 %) | 98 (21.4 %) | 17 (3.7 %) | 8 (1.8 %) | 2 (.4 %) | 2 (.4 %) | 2 (.4 %) | 30 |
| **Constraints** | | | | | | | |  |
| **“Everyday stress prevents me from getting vaccinated.”** | | | | | | | | |
| All | 1,224 (40.4 %) | 761 (25.1 %) | 400 (13.2 %) | 218 (7.2 %) | 335 (11.1 %) | 70 (2.3 %) | 18 (.6 %) | 224 |
| No intention to receive vacc. | 212 (34.6 %) | 158 (25.8 %) | 92 (15.0 %) | 56 (9.2 %) | 72 (11.8 %) | 16 (2.6 %) | 6 (1.0 %) | 45 |
| Intention, not tried to receive vacc. | 428 (33.5 %) | 314 (24.6 %) | 191 (14.9 %) | 100 (7.8 %) | 188 (14.7 %) | 48 (3.8 %) | 9 (.7 %) | 68 |
| Intention, tried to receive vacc. | 301 (44.3 %) | 172 (25.3 %) | 91 (13.4 %) | 47 (6.9 %) | 60 (8.8 %) | 6 (.9 %) | 2 (.3 %) | 41 |
| Vaccinated | 283 (61.9 %) | 117 (25.6 %) | 26 (5.7 %) | 15 (3.3 %) | 15 (3.3 %) | 0 (-) | 1 (.2 %) | 30 |
| **“For me, it is inconvenient to receive vaccinations.”** | | | | | | | | |
| All | 575 (19.0 %) | 562 (18.6 %) | 466 (15.4 %) | 362 (12.0 %) | 535 (17.7 %) | 317 (10.5 %) | 209 (6.9 %) | 224 |
| No intention to receive vacc. | 130 (21.2 %) | 155 (25.3 %) | 104 (17.0 %) | 90 (14.7 %) | 84 (18.5 %) | 36 (13.7 %) | 13 (5.9 %) | 45 |
| Intention, not tried to receive vacc. | 226 (17.7 %) | 224 (17.5 %) | 222 (17.4 %) | 161 (12.6 %) | 263 (20.6 %) | 127 (9.9 %) | 55 (4.3 %) | 68 |
| Intention, tried to receive vacc. | 106 (15.6 %) | 85 (12.5 %) | 71 (10.5 %) | 70 (10.3 %) | 127 (18.7 %) | 111 (16.3 %) | 109 (16.1 %) | 41 |
| Vaccinated | 113 (24.7 %) | 98 (21.4 %) | 69 (15.1 %) | 41 (9.0 %) | 61 (13.3 %) | 43 (9.4 %) | 32 (7.0 %) | 30 |
| **“Visiting the doctor’s makes me feel uncomfortable; this keeps me from getting vaccinated.”** | | | | | | | | |
| All | 1,680 (55.5 %) | 706 (23.3 %) | 246 (8.1 %) | 153 (5.1 %) | 147 (4.9 %) | 66 (2.2 %) | 28 (.9 %) | 224 |
| No intention to receive vacc. | 283 (46.2 %) | 155 (25.3 %) | 53 (8.7 %) | 47 (7.7 %) | 34 (5.6 %) | 29 (4.7 %) | 11 (1.8 %) | 45 |
| Intention, not tried to receive vacc. | 655 (54.6 %) | 302 (23.6 %) | 125 (8.8 %) | 69 (5.0 %) | 93 (5.6 %) | 26 (1.8 %) | 8 (.7 %) | 68 |
| Intention, tried to receive vacc. | 413 (60.8 %) | 159 (23.4 %) | 47 (6.9 %) | 29 (4.3 %) | 16 (2.4 %) | 9 (1.3 %) | 6 (.9 %) | 41 |
| Vaccinated | 329 (72.0 %) | 90 (19.7 %) | 21 (4.6 %) | 8 (1.8 %) | 4 (.9 %) | 2 (.4 %) | 3 (.7 %) | 30 |
| **Calculation** | | | | | | | |  |
| **“When I think about getting vaccinated, I weigh benefits and risks to make the best decision possible.”** | | | | | | | | |
| All | 168 (5.6 %) | 189 (6.2 %) | 204 (6.7 %) | 388 (12.8 %) | 646 (21.3 %) | 906 (29.9 %) | 525 (17.3 %) | 224 |
| No intention to receive vacc. | 16 (2.6 %) | 23 (3.8 %) | 26 (4.2 %) | 63 (10.3 %) | 156 (25.5 %) | 201 (32.8 %) | 127 (20.8 %) | 45 |
| Intention, not tried to receive vacc. | 82 (6.4 %) | 88 (6.9 %) | 105 (8.2 %) | 177 (13.8 %) | 248 (19.4 %) | 375 (29.3 %) | 203 (15.9 %) | 68 |
| Intention, tried to receive vacc. | 40 (5.9 %) | 43 (6.3 %) | 44 (6.5 %) | 89 (13.1 %) | 153 (22.5 %) | 196 (28.9 %) | 114 (16.8 %) | 41 |
| Vaccinated | 30 (6.6 %) | 35 (7.7 %) | 29 (6.3 %) | 59 (12.9 %) | 89 (19.5 %) | 134 (29.3 %) | 81 (17.7 %) | 30 |
| **“For each and every vaccination, I closely consider whether it is useful for me.”** | | | | | | | | |
| All | 149 (4.9 %) | 263 (8.7 %) | 399 (13.2 %) | 401 (13.3 %) | 733 (24.2 %) | 708 (23.4 %) | 372 (12.3 %) | 225 |
| No intention to receive vacc. | 15 (2.5 %) | 30 (4.9 %) | 68 (11.1 %) | 69 (11.3 %) | 158 (25.8 %) | 156 (25.5 %) | 116 (19.0 %) | 45 |
| Intention, not tried to receive vacc. | 77 (6.0 %) | 148 (11.6 %) | 193 (15.1 %) | 186 (14.6 %) | 310 (24.3 %) | 256 (20.0 %) | 107 (8.4 %) | 69 |
| Intention, tried to receive vacc. | 34 (5.0 %) | 52 (7.8 %) | 75 (11.0 %) | 89 (13.1 %) | 173 (25.5 %) | 169 (24.9 %) | 86 (12.7 %) | 41 |
| Vaccinated | 23 (5.0 %) | 32 (7.0 %) | 63 (13.8 %) | 57 (12.5 %) | 92 (20.1 %) | 127 (27.8 %) | 63 (13.8 %) | 30 |
| **“It is important for me to fully understand the topic of vaccination, before I get vaccinated.”** | | | | | | | | |
| All | 61 (2.0 %) | 165 (5.5 %) | 330 (10.9 %) | 378 (12.5 %) | 836 (27.6 %) | 833 (27.5 %) | 422 (14.0 %) | 225 |
| No intention to receive vacc. | 8 (1.3 %) | 24 (3.9 %) | 57 (9.3 %) | 72 (11.8 %) | 179 (29.2 %) | 148 (24.2 %) | 124 (20.3 %) | 45 |
| Intention, not tried to receive vacc. | 35 (2.7 %) | 89 (7.0 %) | 177 (13.9 %) | 160 (12.5 %) | 356 (27.9 %) | 326 (25.5 %) | 134 (10.5 %) | 69 |
| Intention, tried to receive vacc. | 11 (1.6 %) | 36 (5.3 %) | 60 (8.8 %) | 98 (14.4 %) | 179 (26.4 %) | 208 (30.6 %) | 87 (12.8 %) | 41 |
| Vaccinated | 7 (1.5 %) | 16 (3.5 %) | 36 (7.9 %) | 48 (10.5 %) | 122 (26.7 %) | 151 (33.0 %) | 77 (16.8 %) | 30 |
| **Collective Responsibility** | | | | | | | | |
| **“When everyone is vaccinated, I don’t have to get vaccinated, too.” (R)** | | | | | | | | |
| All | 2,086 (69.0 %) | 693 (22.9 %) | 139 (4.6 %) | 66 (2.2 %) | 18 (.6 %) | 10 (.3 %) | 13 (.4 %) | 225 |
| No intention to receive vacc. | 327 (58.5 %) | 175 (28.6 %) | 51 (8.3 %) | 37 (6.0 %) | 11 (1.8 %) | 4 (.7 %) | 7 (1.1 %) | 45 |
| Intention, not tried to receive vacc. | 958 (75.0 %) | 258 (20.2 %) | 44 (3.4 %) | 9 (.7 %) | 4 (.3 %) | 3 (.2 %) | 1 (.1 %) | 69 |
| Intention, tried to receive vacc. | 498 (73.3 %) | 145 (21.4 %) | 23 (3.4 %) | 9 (1.3 %) | 2 (.3 %) | 2 (.3 %) | 0 (-) | 41 |
| Vaccinated | 303 (66.2 %) | 115 (25.1 %) | 21 (4.6 %) | 11 (2.4 %) | 1 (.2 %) | 1 (.2 %) | 5 (1.1 %) | 30 |
| **“I get vaccinated because I can also protect people with a weaker immune system.”** | | | | | | | | |
| All | 46 (1.5 %) | 48 (1.6 %) | 66 (2.2 %) | 141 (4.7 %) | 285 (9.4 %) | 733 (24.2 %) | 1706 (56.4 %) | 225 |
| No intention to receive vacc. | 28 (4.6 %) | 24 (3.9 %) | 24 (3.9 %) | 43 (7.0 %) | 80 (13.1 %) | 166 (27.1 %) | 247 (40.4 %) | 45 |
| Intention, not tried to receive vacc. | 6 (.5 %) | 11 (.9 %) | 11 (.9 %) | 35 (2.7 %) | 76 (6.0 %) | 279 (21.8 %) | 859 (67.3 %) | 69 |
| Intention, tried to receive vacc. | 5 (.7 %) | 7 (1.0 %) | 12 (1.8 %) | 38 (5.6 %) | 69 (10.2 %) | 169 (24.9 %) | 379 (55.8 %) | 41 |
| Vaccinated | 7 (1.5 %) | 6 (1.3 %) | 19 (4.2 %) | 25 (5.5 %) | 60 (13.1 %) | 119 (26.0 %) | 221 (48.4 %) | 30 |
| **“Vaccination is a collective action to prevent the spread of diseases.”** | | | | | | | | |
| All | 34 (1.1 %) | 20 (.7 %) | 15 (.5 %) | 37 (1.2 %) | 135 (4.5 %) | 556 (18.4 %) | 2,228 (73.7 %) | 225 |
| No intention to receive vacc. | 25 (4.1 %) | 11 (1.8 %) | 10 (1.6 %) | 19 (3.1%) | 42 (6.9 %) | 146 (23.9 %) | 359 (58.7 %) | 45 |
| Intention, not tried to receive vacc. | 2 (.2 %) | 2 (.2 %) | 0 (-) | 6 (.5 %) | 36 (2.8 %) | 190 (14.9 %) | 1,041 (81.5 %) | 69 |
| Intention, tried to receive vacc. | 2 (.3 %) | 3 (.4 %) | 1 (.1 %) | 6 (.9 %) | 31 (4.6 %) | 124 (18.3 %) | 512 (75.4 %) | 41 |
| Vaccinated | 5 (1.1 %) | 4 (.9 %) | 4 (.9 %) | 6 (1.3 %) | 26 (5.7 %) | 96 (21.0 %) | 316 (69.2 %) | 30 |

(R): Item is reverse-coded
